# Supplementary material for: Convergent evidence for the temperature-dependent emergence of silicification in terrestrial plants
Source: Nat Commun. 2025 Jan 29;16:1155. doi: 10.1038/s41467-025-56438-0 (PMC11779819; doi:10.1038/s41467-025-56438-0)
Supplement: Supplementary file 1 — Supplementary Information [file 41467_2025_56438_MOESM1_ESM.pdf]

Supplementary materials

Databases	Descriptions	Sources
Plant Si database	Contains leaf Si concentrations of 1826 species	[1]
GBIF	To obtain the geographical distribution of plants	www.gbif.org
CHELSA and RESDC	To obtain the climate variables	chelsa-climate.org www.resdc.cn/DOI
Wheat, rice, weeping willow and winter jasmine phytolith/Si database	475 wheat individuals, 279 rice individuals, 140 weeping willow individuals, and 80 winter jasmine individuals	Field sampling
NCBI BLASTP	To construct phylogenetic trees of Si transporters	blast.ncbi.nlm.nih.gov
Angiosperm phylogeny website	To obtane differentiation time of high-Si and low-Si species	www.mobot.org/MOBOT/research/APweb/
Earth historic temperature database	Contains 5 million years of historical Earth temperature data and division of Earth warming and cooling episodes	[2]

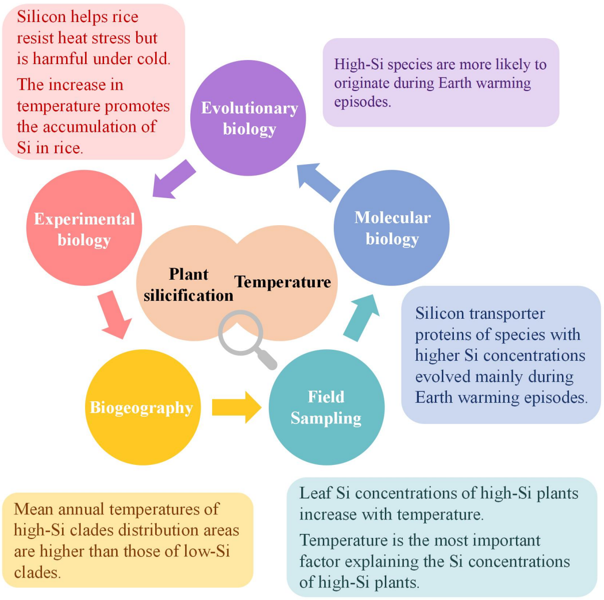

Fig. S1 The introduction of the data used in this paper and the core evidence of the relationship between temperature and plant silicification.

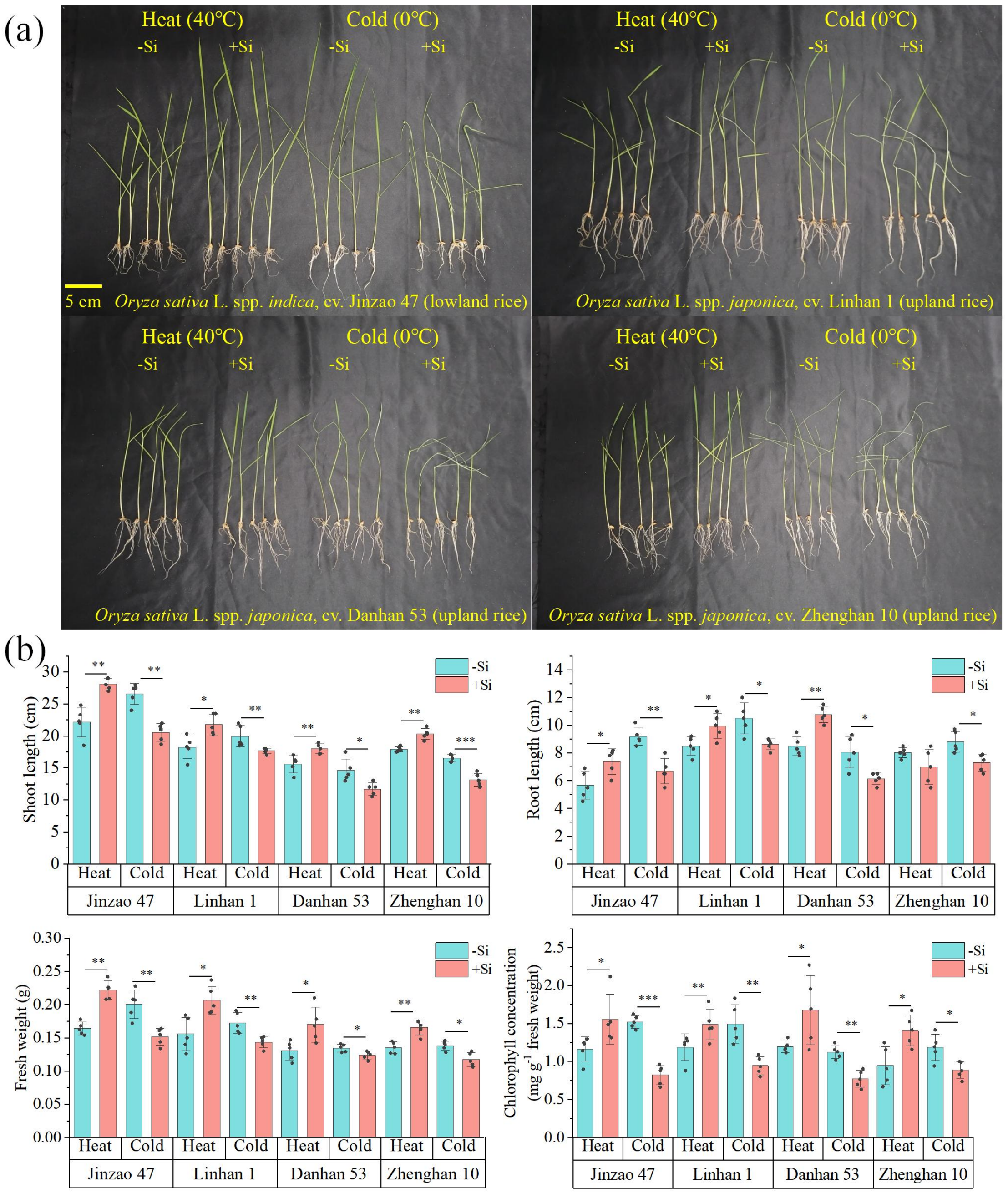

Fig. S2 The effect of Si on rice growth under heat and cold stress. (a) Images of rice after three days of under heat (40°C) and cold (0°C) stress. (b) The shoot length, root length, fresh weight (the sum of shoots and roots) and chlorophyll concentration of rice. The rice (*Oryza sativa*) varieties used in this experiment include spp. *indica*, cv. Jinzao 47 (lowland rice), spp. *japonica*, cv. Linhan 1 (upland rice), spp. *japonica*, cv. Danhan 53 (upland rice), and spp. *japonica*, cv. Zhenghan 10 (upland rice). The +Si group refers to the cultivation of 10-day old rice in a nutrient solution containing 1mM silicic acid for 15 days prior to stress. The data represent the mean and standard deviation of five replicates. Differences between groups are compared using two-sided Welch t-tests. Significant differences between the groups without and with Si are indicated as follows: * $p < 0.05$, ** $p < 0.01$, *** $p < 0.001$.

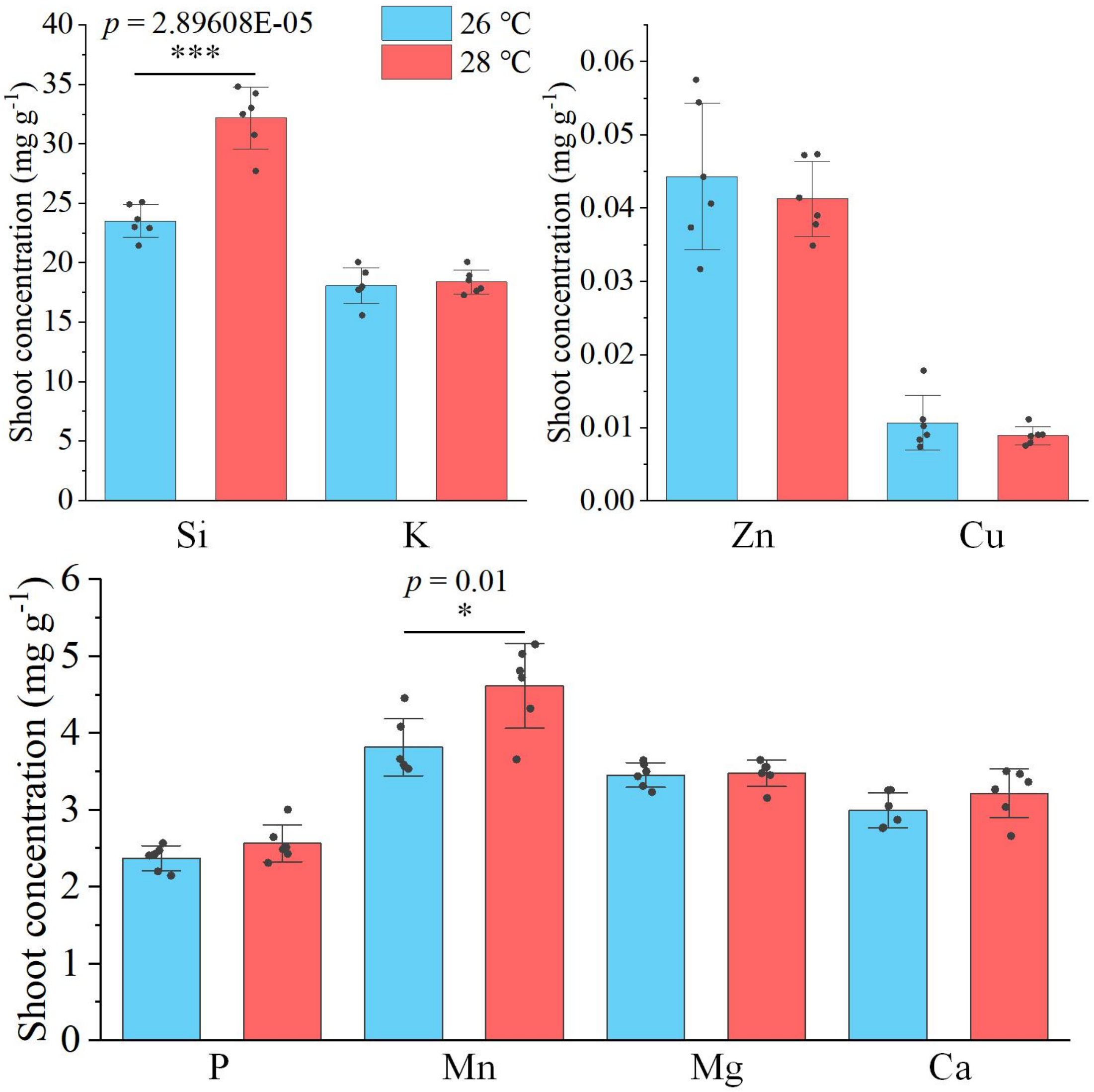

Fig. S3 Concentration of elements in rice leaves cultured at 26 °C and 28 °C for 10 days. The elements include Si, potassium (K), zinc (Zn), copper (Cu), phosphorus (P), manganese (Mn), magnesium (Mg), and calcium (Ca). The data represent the mean and standard deviation of six replicates. Differences between groups are compared using two-sided Welch t-tests. Significant differences are indicated as follows: ** $p < 0.01$, *** $p < 0.001$.

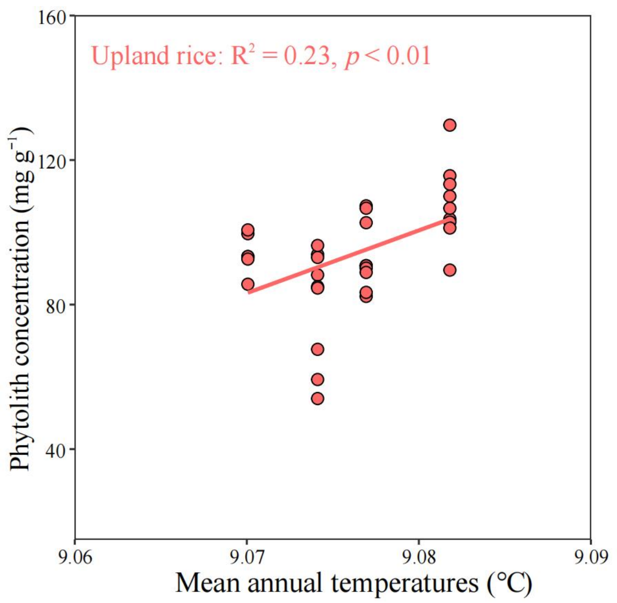

Fig. S4 Correlation analysis of upland rice phytolith concentrations with mean annual temperatures. Leaf phytoliths were extracted from dried leaves by microwave digestion, and then dried and weighed. The samples of upland rice are from Xinjiang (n = 33 individuals). The Student's t-test for the regression coefficient is two-sided ($p = 0.005$).

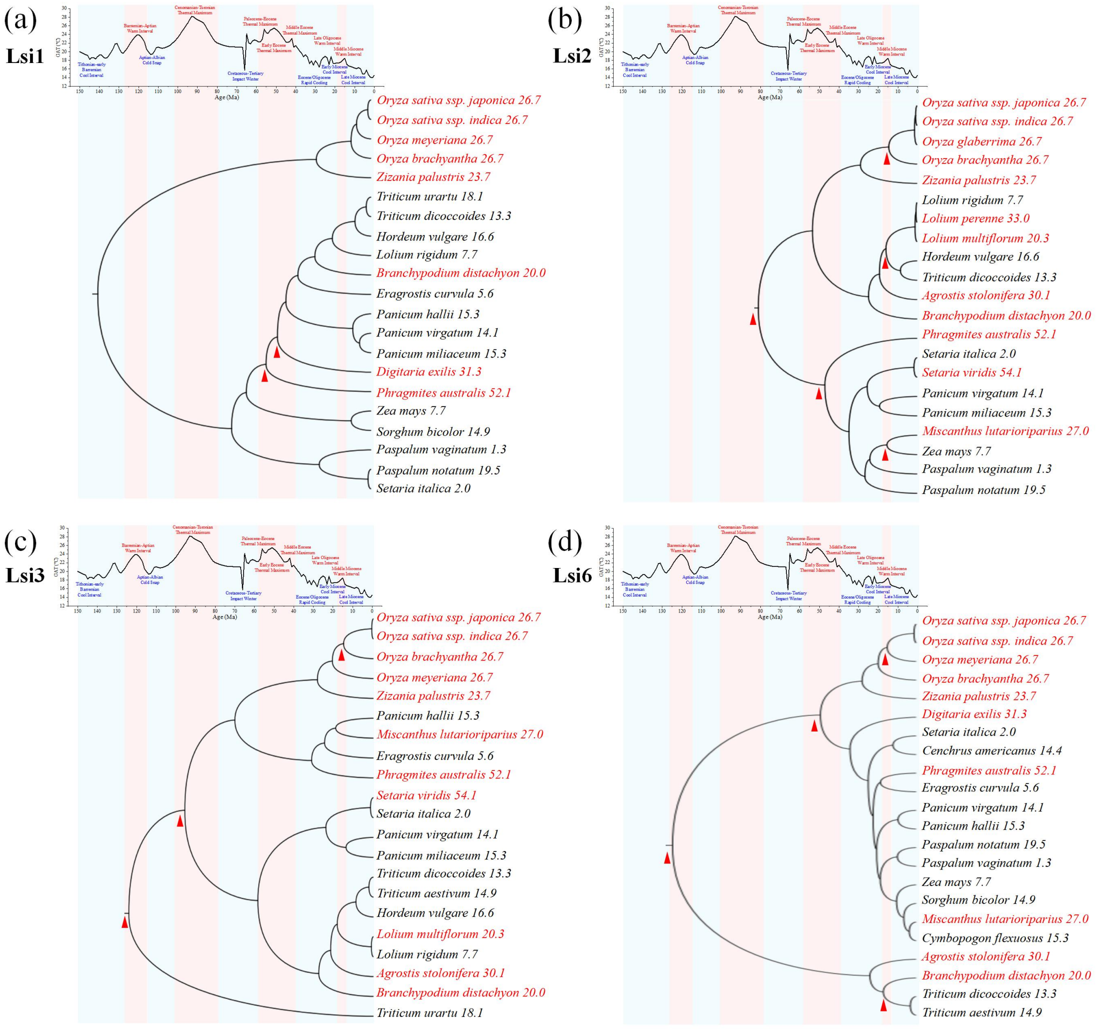

Fig. S5 Evolutionary relationships between Si uptake and transport proteins within the grasses (Poaceae). (a-d) represent Lsi1, Lsi2, Lsi3, and Lsi6, respectively. Homologous sequences of Si uptake and transport proteins in *Oryza sativa* ssp. *japonica* are searched by BLASTP. Protein sequences from the top 20 species with the highest homology are selected for the analysis. Sequence alignment is performed using MEGA11. The Jones-Taylor-Thornton (JTT) model is selected as the most suitable amino acid substitution model with Gamma-distributed rates among sites using Prottest (version 3.4.2). The corresponding timetree is constructed via Phylogenetic Analysis by Maximum Likelyhood (PAML). The differentiation time of Lsi proteins is estimated by MCMCtree, a program applying the Bayesian Markov Chain Monte Carlo (MCMC) method, and checked by the pairwise divergence time of 3 couples of species provided by Timetree. The leaf Si concentration of the species follows its name (unit in mg g^{-1}), and the data is from de Tombeur (2023)[1]. The upper part of each graph shows the changes in Earth's average temperature from 150 Ma to the present. The red area represents the warming episodes, and the blue area represents the cooling episodes. The division is based on Scotese (2021)[3]. The results indicate that the silicon absorption and transport proteins of species with higher Si concentrations (red font, leaf Si concentration > 20 mg g^{-1}) mainly differentiate during the warming episodes (red arrows).

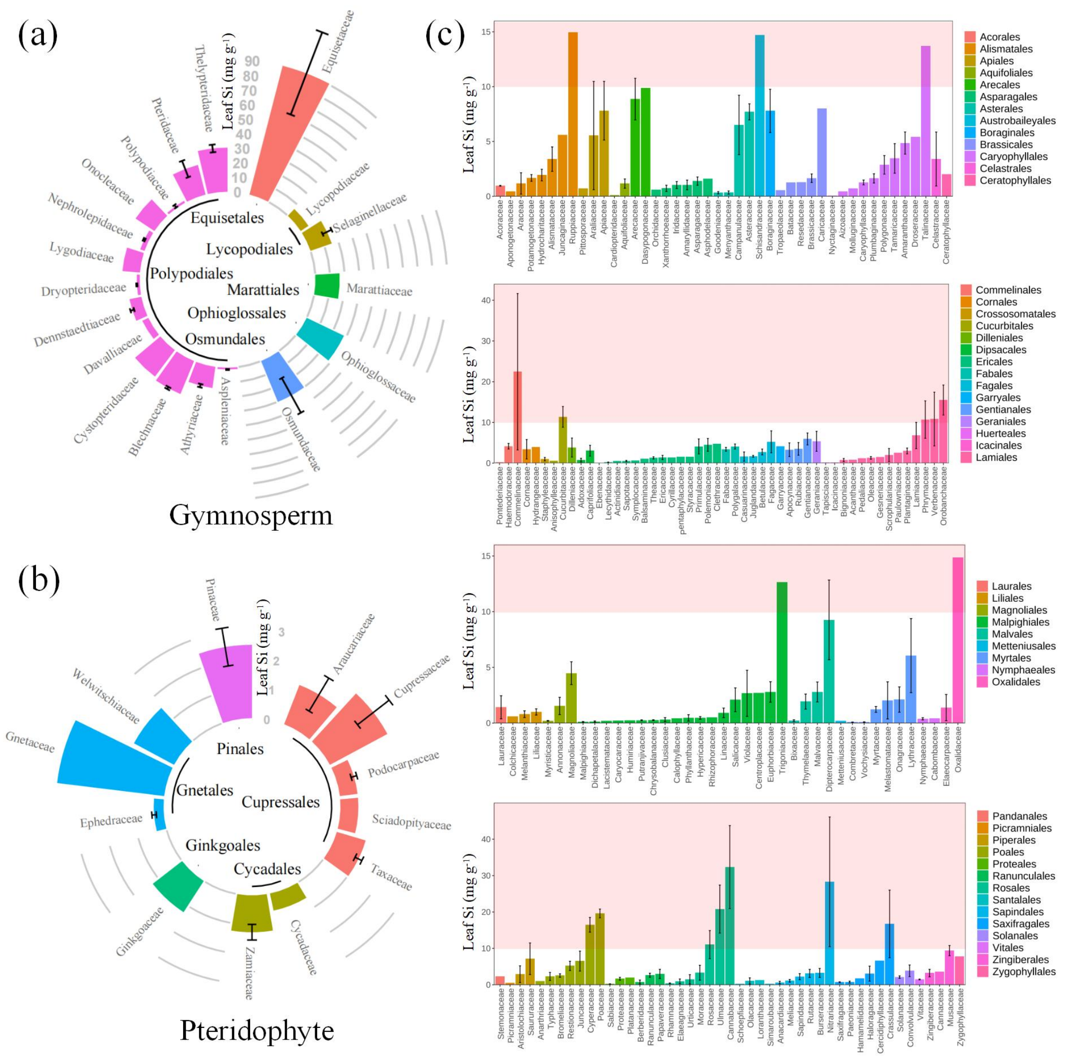

Fig. S6 Large variation in leaf Si concentrations within the plant kingdom. Leaf Si concentrations of **(a)** gymnosperms and **(b)** pteridophytes. Each column represents a family and the same color represents the same order. (**c**) Leaf Si concentrations of plants from different families of angiosperms. Each column represents a family and the same color represents the same order. Orders are displayed in alphabetical order. Red areas indicate Si concentrations higher than 10 mg g^{-1}. Error bars indicate standard errors. The data can be obtained from de Tombeur *et al.*[1], as shown in Supplementary Data 2. In this paper, we classify species with Si concentrations higher than 10 mg g^{-1} as high-Si species, and species with Si concentrations lower than 1 mg g^{-1} as low-Si species. By analyzing the data from de Tombeur *et al.*[1], we find that pteridophytes generally have high Si concentrations (n = 44 species), with 54.5% of pteridophytes containing ＞10 mg g^{-1} Si and only 9.1% containing <1 mg g^{-1} Si. In contrast, the Si concentration of gymnosperms is very low (n = 83 species), with 55.4% of gymnosperms containing ＜1 mg g^{-1} Si and only 2.4% containing >10 mg g^{-1} Si (Fig. S6a, b). Among Angiosperms (n = 181 families), around 9% of families have a mean Si concentration higher than 10 mg g^{-1} while 32% of the families have a mean Si concentration lower than 1 mg g^{-1}. The 17 families of angiosperms with high leaf Si concentrations >10 mg g^{-1} rang from 32.4 mg g^{-1} (Cannabaceae) to 10.7 mg g-1 (Phrymaceae), with an average of 16.9 ± 6.2 mg g^{-1}. A total of 58 families with low leaf Si concentrations <1 mg g^{-1} are dispersed in 31 orders with an average of 0.5 ± 0.3 mg g^{-1} (Fig. S6c).

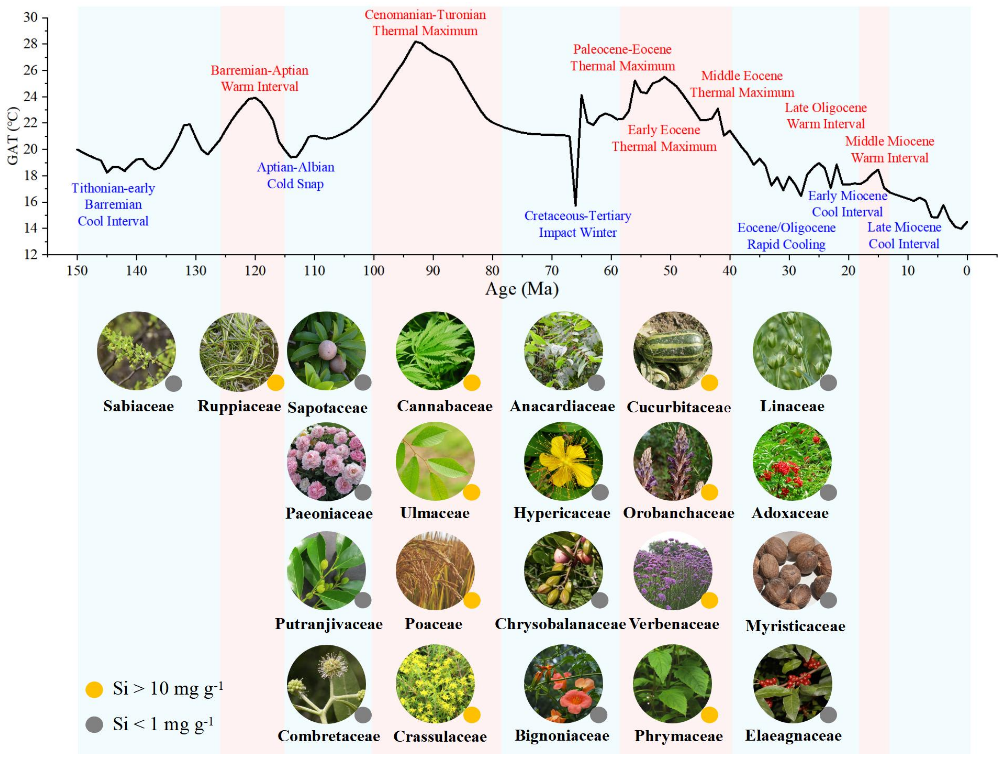

Fig. S7 Link between global average temperature (GAT) in history and the emergence of high-Si vs. low-Si families. The line chart represents the change in the average temperature of the Earth from 150 Ma to the present, with red areas indicating warming episodes and blue areas indicating cooling episodes, and the division is based on Scotese *et al.*[3]. Plant icons indicate typical families that emerged in the corresponding geological periods. Families with Si concentrations above 10 mg g^{-1} are marked orange and families with Si concentrations below 1 mg g^{-1} are marked grey. Phylogenetic data are provided by Angiosperm Phylogeny Website (www.mobot.org/MOBOT/research/APweb/).

(a)

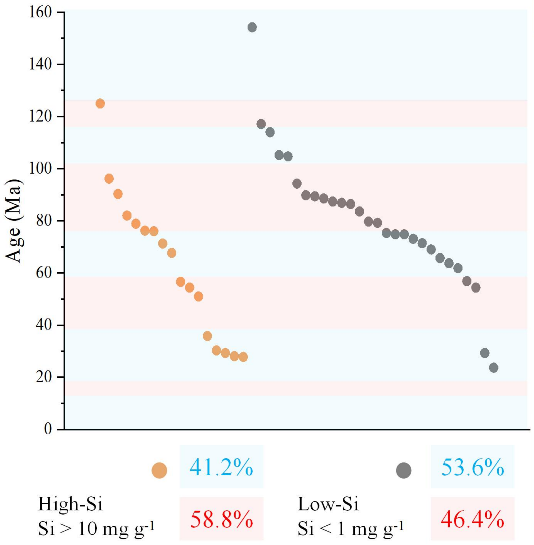

(b)

High-Si	Age (Ma)	Low-Si	Age (Ma)	Low-Si	Age (Ma)
Schisandraceae	125	Acoraceae	154.2	Elaeagnaceae	74.8
Crassulaceae	96.2	Myristicaceae	117.1	Bixaceae	73.1
Rosaceae	90.3	Nymphaeaceae	114	Sapotaceae	71.4
Commelinaceae	82	Paeoniaceae	105.2	Adoxaceae	69
Cyperaceae	78.9	Sabiaceae	104.7	Chrysobalanaceae	65.7
Ulmaceae	76.2	Putranjivaceae	94.3	Malpighiaceae	63.7
Poaceae	76	Clusiaceae	89.8	Anacardiaceae	61.8
Nitrariaceae	71.3	Combretaceae	89.4	Saxifragaceae	56.9
Cannabaceae	67.7	Lecythidaceae	88.6	Dichapetalaceae	54.4
Cucurbitaceae	56.6	Berberidaceae	87.4	Bignoniaceae	29.3
Trigoniaceae	54.4	Linaceae	86.9	Staphyleaceae	23.7
Oxalidaceae	51	Hypericaceae	86.4		
Ruppiaceae	35.8	Vochysiaceae	83.6		
Talinaceae	30.3	Menyanthaceae	79.7		
Verbenaceae	29.3	Melanthiaceae	79.2		
Orobanchaceae	28.1	Goodeniaceae	75.3		
Phrymaceae	27.8	Rhamnaceae	74.8		

Fig. S8 Emergence of plant families with different Si strategies during warming and cooling episodes. Evolutionary information was obtained from the time-calibrated plastid phylogenomic tree in Li et al.'s paper[4]. The results show that 58.8% of families with high Si emerged during the warming episodes, and 53.6% of families with low Si emerged during the cooling episodes. Each point represents a family. Families with Si concentration above 10 mg g^{-1} are marked orange, and families with Si concentration below 1 mg g^{-1} are marked gray. Red areas indicate warming episodes and blue areas indicate cooling episodes, and the division is based on Scotese *et al.*[3].

Table S1 The growth status of rice in +Si and -Si groups before stress. There is no significant difference in growth indicators between +Si and -Si groups before stress (n = 5, two-sided Welch t-test).

Before stress	-Si	+Si	*p* value
Shoot length (cm)	30.62±1.45	32.18±2.27	0.23
Root length (cm)	13.59±1.7	13.15±1.62	0.68
Fresh weight (g)	0.89±0.05	0.93±0.04	0.16
Chlorophyll concentration (mg g^{-1} fresh weight)	2.83±0.28	2.68±0.16	0.20

Table S2 Multiple regression results of climate variables and Si concentration in leaves of wheat and rice.

Wheat	Regression results				
Variables	Coefficient	95% Confidence Interval	Standard error	t-statistic	*p* value
Intercept	0	-0.08 – 0.08	0.04	0	1
MAT	0.69 *	0.07 – 1.32	0.32	2.18	0.03
GST	-0.2	-0.83 – 0.43	0.32	-0.61	0.54
EVP	0.25	-0.13 – 0.64	0.2	1.29	0.199
PRE	-0.14	-0.31 – 0.03	0.09	-1.59	0.112
PRS	-0.18 **	-0.29 – -0.06	0.06	-2.89	0.004
RHU	0.22	-0.07 – 0.51	0.15	1.49	0.138
WIN	-0.02	-0.16 – 0.12	0.07	-0.33	0.745
RSDS	-0.13	-0.26 – 0.00	0.07	-1.91	0.057
VPD	-0.1	-0.35 – 0.16	0.13	-0.73	0.467
Observations	475				
R^2 / R^2 adjusted	0.215 / 0.199				
Akaike information criterion	1254.223				
p value	< 2.2e-16				
					* *p<0.05* ** *p<0.01* *** *p<0.001*

Rice	Regression results				
Variables	Coefficient	95% Confidence Interval	Standard error	t-statistic	*p* value
Intercept	0	-0.09 – 0.09	0.05	0	1

MAT	5.57 ***	3.60 – 7.54	1	5.57	6.78e-08
GST	-3.92 ***	-5.74 – -2.10	0.92	-4.24	3.19e-05
EVP	-0.55 ***	-0.83 – -0.27	0.14	-3.88	0.0001
PRE	-0.11	-0.30 – 0.07	0.09	-1.21	0.227
PRS	0	-0.11 – 0.11	0.06	-0.02	0.984
RHU	-0.34 ***	-0.47 – -0.21	0.07	-5.16	5.32e-07
WIN	-0.25 **	-0.43 – -0.07	0.09	-2.77	0.006
RSDS	0.13 *	0.00 – 0.25	0.06	2.04	0.043
VPD	-1.26 ***	-1.77 – -0.76	0.26	-4.93	1.52e-06
Observations	246				
R^2 / R^2 adjusted	0.506 / 0.488				
Akaike information criterion	545.456				
p value	< 2.2e-16				
					* *p<0.05* ** *p<0.01* *** *p<0.001*

Table S3 The pairwise divergence time used to construct the timetree for each protein obtained from Timetree (https://timetree.org/). Three couples of species divergence times are used in the evolutionary tree of each protein.

Protein	Species 1	Species 2	Divergence time (Ma)
Lsi1	*Zea mays*	*Sorghum bicolor*	9-11.8
	Hordeum vulgare	*Triticum dicoccoides*	6.8-18.3
	Zizania palustris	*Oryza sativa*	21.9-31.2
Lsi2	*Setaria_italica*	*Setaria_viridis*	0.7-2.1
	Hordeum vulgare	*Triticum dicoccoides*	6.8-18.3
	Zizania palustris	*Oryza sativa*	21.9-31.2
Lsi3	*Setaria_italica*	*Setaria_viridis*	0.7-2.1
	Hordeum vulgare	*Triticum dicoccoides*	6.8-18.3
	Zizania palustris	*Oryza sativa*	21.9-31.2
Lsi6	*Zea mays*	*Sorghum bicolor*	9-11.8
	Zizania palustris	*Oryza sativa*	21.9-31.2
	Triticum_dicoccoides	*Triticum_aestivum*	0.432

Table S4 Leaf Si concentrations and differentiation times in high- (> 10 mg g^{-1}) and low-Si (< 1 mg g^{-1}) angiosperm families. Leaf Si concentration information comes from de Tombeur et al. (2023)[1]. Historical climate change information comes from Scotese et al.[2].

Type	Families	Leaf Si concentrations	Differentiation times (Ma)	Temperatures (℃)
High-Si	Ruppiaceae	14.96	117.5	22.6
High-Si	Nitrariaceae	28.30	96.5	25.7
High-Si	Rosaceae	11.04	88.3	27
High-Si	Cyperaceae	16.68	87.8	27
High-Si	Cannabaceae	32.36	87.4	26.7
High-Si	Ulmaceae	20.80	85.4	25.2
High-Si	Poaceae	19.60	83	23.7
High-Si	Crassulaceae	16.73	81.7	22.9
High-Si	Commelinaceae	22.45	62	22.5
High-Si	Cucurbitaceae	11.37	59	22.3
High-Si	Oxalidaceae	14.86	52	25.2
High-Si	Orobanchaceae	15.50	50.4	25.1
High-Si	Verbenaceae	10.83	42.6	22.4
High-Si	Schisandraceae	14.70	42.2	23.1
High-Si	Phrymaceae	10.69	40	21.4
High-Si	Trigoniaceae	12.65	31.6	17.9
High-Si	Talinaceae	13.71	29.9	17.9
Low-Si	Sabiaceae	0.21	129	20.0
Low-Si	Berberidaceae	0.75	123.7	22.2
Low-Si	Nymphaeaceae	0.38	121	23.8
Low-Si	Sapotaceae	0.53	107	20.9
Low-Si	Paeoniaceae	0.75	106	21.1
Low-Si	Putranjivaceae	0.25	105	21.3
Low-Si	Combretaceae	0.07	102.6	21.9
Low-Si	Melanthiaceae	0.80	97	25.3
Low-Si	Anacardiaceae	0.60	72.7	21.2
Low-Si	Hypericaceae	0.47	71.5	21.1
Low-Si	Goodeniaceae	0.32	67.3	21
Low-Si	Chrysobalanaceae	0.26	66.2	15.7
Low-Si	Lecythidaceae	0.22	65	24.1
Low-Si	Clusiaceae	0.31	63.6	22.1
Low-Si	Menyanthaceae	0.34	63.4	21.9
Low-Si	Rhamnaceae	0.40	62	22.5
Low-Si	Saxifragaceae	0.69	61.8	22.5
Low-Si	Bignoniaceae	0.77	51.5	25.2
Low-Si	Vochysiaceae	0.09	39	20.8
Low-Si	Linaceae	0.92	36	18.8

Low-Si	Malpighiaceae	0.11	36	18.8
Low-Si	Bixaceae	0.21	34.3	18.8
Low-Si	Adoxaceae	0.75	33	17.3
Low-Si	Myristicaceae	0.20	32.6	17.3
Low-Si	Staphyleaceae	0.99	26	18.6
Low-Si	Dichapetalaceae	0.12	20.7	17.4
Low-Si	Elaeagnaceae	0.95	20	17.3
Low-Si	Acoraceae	0.96	19	17.4

Table S5 Si concentrations and differentiation times in the lower taxonomic units of the five major angiosperms families. In addition to the information labelled with new references, the silicon concentration information comes from de Tombeur *et al.*[1].

Families	Lower taxonomic units	Si concentrations	Differentiation times (Ma)	Temperatures
Asteraceae	Asteroideae	High	43	Warm
	Carduoideae	Low	34.1	Cool
	Cichorioideae	Low	23.5	Cool
Orchidaceae	Apostasioideae	High	47	Warm
	Vanilloideae	Low	68	Cool
	Cypripedioideae	High	46.5	Warm
	Orchidoideae	Low	55	Cool
	Epidendroideae	Low	55	Cool
Fabaceae	Caesalpinioideae	Low	63.4	Cool
	Cercidoideae	Low	34	Cool
	Detarioideae	Low	51.9	Cool
	Dialioideae	Low	34	Cool
	Papilionoideae	Low	63.8	Cool
	Dalbergieae	Low	50.7	Warm
	Fabeae	Low	22.1	Cool
	Genisteae	Low	32.3	Cool
	Mirbelieae	Low	48.4	Warm
	Ormosieae	Low	40.8	Cool
	Sophoreae	Low	40.8	Cool
	Trifolieae	Low	36	Cool
	Phaseoleae	High	55.2	Warm
Rubiaceae	Rubioideae	High	84.4	Warm
	Ixoroideae	Low	73.1	Cool
	Cinchonoideae	Low	73.1	Cool
Poaceae	Poodinae	Low	62.1	Cool
	Panicoideae	Low	23.6	Cool
	Poeae	Low	28.8	Cool
	Chloridoideae	Low	54.7	Cool
	Paspaleae	Low	36	Cool
	Arundinarieae	High	14.3	Warm
	Panicoideae	High	23.6	Warm
	Cynodonteae	High	32.7	Cool
	Molinieae	High	7.1	Warm

a) Asteraceae (n = 142 species): A great variation in Si was observed within the Asteraceae. 90.2% high-Si species (>10 mg g^{-1}) belong to the Asteroideae subfamily, which emerged at ~43 Ma[5,6] during the Middle Eocene Thermal Maximum. In contrast, the subfamilies Carduoideae (34.1 Ma)[7] and Cichorioideae (23.5 Ma)[8], which emerged during the Early Oligocene Cooling

Event and the Early Miocene Cool Interval, have a large number of species that contain less than 2 mg g^{-1} Si.

b) Orchidaceae (n = 81): Within the Orchidaceae, the Apostasioideae (47 Ma) and Cypripedioideae (46.5 Ma) subfamilies emerged during the Paleocene-Eocene Hothouse and exhibit high Si concentrations[9,10]. By contrast, the Vanilloideae, Orchidoideae, and Epidendrioideae, which emerged during the Late Cretaceous-Paleocene Cooling Interval, are virtually free of Si[10].

c) Fabaceae (n = 152): Species in the Fabaceae with low Si concentrations <1 mg g^{-1} only belong to the Caesalpinioideae, Cercidoideae, Detarioideae, Dialioideae, and Papilionoideae subfamilies, which all emerged during cooling episodes (Late Cretaceous–Paleocene Cool Interval, Early Oligocene Cooling Event). 84.2% of high-Si species (>10 mg g^{-1}) are found in the Papilionoideae. We then further investigated the Si concentrations and divergence times in different clades within the Papilionoideae. Low-Si species mostly belong to the Dalbergieae (50.7 Ma), Fabeae (22.1 Ma), Genisteae (32.3 Ma), Mirbelieae (48.4 Ma), Ormosieae (40.8 Ma), Sophoreae (40.8 Ma), and Trifolieae (36 Ma) (the differentiation times of Fabaceae subfamilies were obtained from Angiosperm Phylogeny Website), with five of them emerging during Late Eocene–Oligocene Cooling. High-Si species belong to the Phaseoleae (55.2 Ma), which emerged during the Early Eocene Thermal Maximum[11,12].

d) Rubiaceae (n = 24): High-Si species of Rubiaceae are mainly found in the Rubioideae, which emerged at approximately 84.4 Ma during Cenomanian–Turonian Thermal Maximum, with a record-high global mean temperature of 28.2°C. Low-Si species are mainly found in the Ixoroideae and Cinchonoideae, which all emerged at 73.1 Ma during the Cretaceous-Tertiary Impact Winter period, when the global mean temperature was only 8.4°C[13].

e) Poaceae (n = 156): Although most Poaceae are typical Si accumulating plants, there are large variations in Si concentration among different phylogenetic groups. Species with Si concentrations <5 mg g^{-1} are only distributed among five subfamilies (Poodinae [62.1 Ma], Panicoideae [23.6 Ma], Poeae [28.8 Ma], Chloridoideae [54.7 Ma], and Paspaleae [36 Ma]) (the differentiation times of Poaceae subfamilies were obtained from Angiosperm Phylogeny Website), with all but Chloridoideae emerging during cooling episodes of the Late Cretaceous–Paleocene Cooling Interval and the Late Eocene–Oligocene Cooling. By contrast, species with high Si concentrations >50 mg g^{-1} mainly belong to the Arundinarieae (14.3 Ma), Panicoideae (23.6 Ma), Cynodonteae (32.7 Ma), and Molinieae (7.1 Ma), of which all but the Cynodonteae emerged during warming episodes (Late Oligocene Warming Interval, Mid-Miocene Thermal Maximum, and Pliocene Warming Interval).

References

1. de Tombeur, F. et al. Why do plants silicify? *Trends Ecol. Evol.* **38**, 275-288 (2023). https://doi.org/10.1016/j.tree.2022.11.002.
2. Scotese, C. R., Song, H., Mills, B. J. W. & van der Meer, D. G. Phanerozoic paleotemperatures: The earth's changing climate during the last 540 million years. *Earth-Sci. Rev.* **215**, 103503 (2021). https://doi.org/10.1016/j.earscirev.2021.103503.
3. Scotese, C. R. An Atlas of Phanerozoic Paleogeographic Maps: The Seas Come In and the Seas Go Out. *Annu. Rev. Earth Planet. Sci.* 49, 679-728 (2021). https://doi.org/10.1146/annurev-earth-081320-064052.
4. Li, H. et al. Origin of angiosperms and the puzzle of the Jurassic gap. *Nat. Plants* **5**, 461-470 (2019). https://doi.org/10.1038/s41477-019-0421-0.
5. Bergh, N. G. & Peter Linder, H. Cape diversification and repeated out-of-southern-Africa dispersal in paper daisies (Asteraceae–Gnaphalieae). *Mol. Phylogenet. Evol.* **51**, 5-18 (2009). https://doi.org/10.1016/j.ympev.2008.09.001.
6. Panero, J. L. & Crozier, B. S. Macroevolutionary dynamics in the early diversification of Asteraceae. *Mol. Phylogenet. Evol.* **99**, 116-132 (2016). https://doi.org/10.1016/j.ympev.2016.03.007.
7. Herrando-Moraira, S. et al. Nuclear and plastid DNA phylogeny of tribe Cardueae (Compositae) with Hyb-Seq data: A new subtribal classification and a temporal diversification framework. *Mol. Phylogenet. Evol.* **137**, 313-332 (2019). https://doi.org/10.1016/j.ympev.2019.05.001.
8. Mandel, J. R. et al. A fully resolved backbone phylogeny reveals numerous dispersals and explosive diversifications throughout the history of Asteraceae. *Proceedings of the National Academy of Sciences* **116**, 14083-14088 (2019). https://doi.org/10.1073/pnas.1903871116.
9. Ramírez, S. R., Gravendeel, B., Singer, R. B., Marshall, C. R. & Pierce, N. E. Dating the origin of the Orchidaceae from a fossil orchid with its pollinator. *Nature* **448**, 1042-1045 (2007). https://doi.org/10.1038/nature06039.
10. Prychid, C. J., Rudall, P. J. & Gregory, M. Systematics and Biology of Silica Bodies in Monocotyledons. *The Botanical review* **69**, 377-440 (2003). https://doi.org/10.1663/0006-8101(2004)069[0377:SABOSB]2.0.CO;2.
11. Lavin, M., Herendeen, P. S. & Wojciechowski, M. F. Evolutionary Rates Analysis of Leguminosae Implicates a Rapid Diversification of Lineages during the Tertiary. *Syst. Biol.* **54**, 575-594 (2005). https://doi.org/10.1080/10635150590947131.
12. Zhao, Y. et al. Nuclear phylotranscriptomics and phylogenomics support numerous polyploidization events and hypotheses for the evolution of rhizobial nitrogen-fixing symbiosis in Fabaceae. *Mol. Plant.* **14**, 748-773 (2021). https://doi.org/10.1016/j.molp.2021.02.006.
13. Bremer, B. & Eriksson, T. Time Tree of Rubiaceae: Phylogeny and Dating the Family, Subfamilies, and Tribes. *Int. J. Plant Sci.* **170**, 766-793 (2009). https://doi.org/10.1086/599077.
