# Supplementary material for: Convergent evidence for the temperature-dependent emergence of silicification in terrestrial plants
Source: Nat Commun. 2025 Jan 29;16:1155. doi: 10.1038/s41467-025-56438-0 (PMC11779819; doi:10.1038/s41467-025-56438-0)
Supplement: Supplementary file 3 — Reporting Summary [file 41467_2025_56438_MOESM3_ESM.pdf]

nature portfolio

Corresponding author(s): Yongchao Liang, Nina Nikolic

Last updated by author(s): Nov 16, 2024

Reporting Summary

Nature Portfolio wishes to improve the reproducibility of the work that we publish. This form provides structure for consistency and transparency in reporting. For further information on Nature Portfolio policies, see our Editorial Policies and the Editorial Policy Checklist.

Statistics

For all statistical analyses, confirm that the following items are present in the figure legend, table legend, main text, or Methods section.

n/a	Confirmed	
☐	☒	The exact sample size (*n*) for each experimental group/condition, given as a discrete number and unit of measurement
☐	☒	A statement on whether measurements were taken from distinct samples or whether the same sample was measured repeatedly
☐	☒	The statistical test(s) used AND whether they are one- or two-sided<br>*Only common tests should be described solely by name; describe more complex techniques in the Methods section.*
☐	☒	A description of all covariates tested
☐	☒	A description of any assumptions or corrections, such as tests of normality and adjustment for multiple comparisons
☐	☒	A full description of the statistical parameters including central tendency (e.g. means) or other basic estimates (e.g. regression coefficient) AND variation (e.g. standard deviation) or associated estimates of uncertainty (e.g. confidence intervals)
☐	☒	For null hypothesis testing, the test statistic (e.g. *F*, *t*, *r*) with confidence intervals, effect sizes, degrees of freedom and *P* value noted<br>*Give P values as exact values whenever suitable.*
☒	☐	For Bayesian analysis, information on the choice of priors and Markov chain Monte Carlo settings
☒	☐	For hierarchical and complex designs, identification of the appropriate level for tests and full reporting of outcomes
☐	☒	Estimates of effect sizes (e.g. Cohen's *d*, Pearson's *r*), indicating how they were calculated

Our web collection on statistics for biologists contains articles on many of the points above.

Software and code

Policy information about availability of computer code

Data collection	Wheat, rice, weeping willow and winter jasmine were collected across China.<br>Climate variables of sampling points were from Resource and Environmental Science Data Center (RESDC, http://www.resdc.cn/DOI) and Earth's Land Surface Areas (CHELSA, https://chelsa-climate.org/).<br>The leaf silicon concentrations of different species and families were obtained from data published by de Tombeur et al. (2023) in Trends in Ecology & Evolution (https://doi.org/10.1016/j.tree.2022.11.002). Taxonomic information was corrected using the NCBI database (www.ncbi.nlm.nih.gov).<br>Homologous sequences of proteins related to Si transport were searched by BLASTP on NCBI (https://blast.ncbi.nlm.nih.gov).<br>The dataset of historic climate change over the last 200 Ma was obtained from the literature in Annual Review of Earth and Planetary Sciences and Earth-Science Reviews (https://doi.org/10.1146/annurev-earth-081320-064052, https://doi.org/10.1016/j.earscirev.2021.103503).<br>The evolutionary time data for both high- and low-Si families were obtained from the Angiosperm Phylogeny Website. (www.mobot.org/MOBOT/research/APweb/) and literature in Nature Plants (https://doi.org/10.1038/s41477-019-0421-0).
Data analysis	The Welch two-sample t-tests and Wilcoxon rank sum test were done using R 4.3.1.<br>We first analyzed the correlation between MAT and leaf phytolith/Si concentration using R 4.3.1. Then, multiple regression models were implemented using ordinary least squares (OLS) in R. In addition, we also assess the relative importance of various climate variables in explaining the phytolith concentration in wheat and rice leaves by using the squared standardized coefficients (betasq) method of relaimpo package in R 4.3.1.<br>The maps were drawn using R 4.3.1.<br>A Maximum-Likelihood phylogenetic tree was constructed using MEGA11 with most suitable amino acid substitution model selected via ProtTest (version 3.4.2). The corresponding timetree was constructed via Phylogenetic Analysis by Maximum Likelihood (PAML), with differentiation time of Lsi proteins estimated by MCMCtree, a program applying the Bayesian Markov Chain Monte Carlo (MCMC) method,

and checked by pairwise divergence time of 3 couples of species provided by Timetree (https://timetree.org/).
The bar charts and box plots were drawn using Origin 2021.

For manuscripts utilizing custom algorithms or software that are central to the research but not yet described in published literature, software must be made available to editors and reviewers. We strongly encourage code deposition in a community repository (e.g. GitHub). See the Nature Portfolio guidelines for submitting code & software for further information.

Data

Policy information about availability of data

All manuscripts must include a data availability statement. This statement should provide the following information, where applicable:

- Accession codes, unique identifiers, or web links for publicly available datasets
- A description of any restrictions on data availability
- For clinical datasets or third party data, please ensure that the statement adheres to our policy

All data generated or used during the study appears in the submitted article.

Research involving human participants, their data, or biological material

Policy information about studies with human participants or human data. See also policy information about sex, gender (identity/presentation), and sexual orientation and race, ethnicity and racism.

Reporting on sex and gender	This study did not involve sex and gender.
Reporting on race, ethnicity, or other socially relevant groupings	This study did not involve race, ethnicity, or other socially relevant groupings.
Population characteristics	This study did not involve population characteristics
Recruitment	This study did not involve human participants.
Ethics oversight	This study did not involve human participants.

Note that full information on the approval of the study protocol must also be provided in the manuscript.

Field-specific reporting

Please select the one below that is the best fit for your research. If you are not sure, read the appropriate sections before making your selection.

☐ Life sciences ☐ Behavioural & social sciences ☒ Ecological, evolutionary & environmental sciences

For a reference copy of the document with all sections, see nature.com/documents/nr-reporting-summary-flat.pdf

Ecological, evolutionary & environmental sciences study design

All studies must disclose on these points even when the disclosure is negative.

Study description	We first investigated the effect of Si on rice (each treatment has five repetitions), a model Si-accumulating species, when grown under high and low temperature stress, and tested the effect of temperature increase on leaf Si concentration of rice, disentangling temperature and transpiration by controlling the humidity (each treatment has six repetitions). We then analyzed whether the global distribution of present-day high- (n=9,972 occurrences) and low-Si (n=9,990 occurrences) clades is related to temperatures in different regions. We also used samples from high-Si (wheat and rice) and low-Si (weeping willow and winter jasmine) species collected along climatic gradients across China, to test Si intraspecific variation in responses to air temperature (wheat, n = 475 individuals; rice, n = 279 individuals; weeping willow, n = 140 individuals; winter jasmine, n=80 individuals). Finally, we analyzed the relationship between the Earth's historical temperature and the periods when high- and low-Si families differentiated.
Research sample	Wheat, rice, weeping willow and winter jasmine were collected across China.<br>Climate variables of sampling points were from Resource and Environmental Science Data Center (RESDC, http://www.resdc.cn/DOI) and Earth's Land Surface Areas (CHELSA, https://chelsa-climate.org/).<br>The leaf silicon concentrations of different species and families were obtained from data published by de Tombeur et al. (2023) in Trends in Ecology & Evolution (https://doi.org/10.1016/j.tree.2022.11.002). Taxonomic information was corrected using the NCBI database (www.ncbi.nlm.nih.gov).<br>Homologous sequences of proteins related to Si transport were searched by BLASTP on NCBI (https://blast.ncbi.nlm.nih.gov).<br>The dataset of historic climate change over the last 200 Ma was obtained from the literature in Annual Review of Earth and Planetary Sciences and Earth-Science Reviews (https://doi.org/10.1146/annurev-earth-081320-064052, https://doi.org/10.1016/j.earscirev.2021.103503).<br>The evolutionary time data for both high- and low-Si families were obtained from the Angiosperm Phylogeny Website. (www.mobot.org/MOBOT/research/APweb/) and literature in Nature Plants (https://doi.org/10.1038/s41477-019-0421-0).

Sampling strategy	For the analysis of distribution temperature, from Hodson et al. (2005), we selected the top ten plant clades with the highest Si concentrations (high-Si clades) and the bottom ten clades with the lowest Si concentrations (low-Si clades). Then, the location of 1000 occurrences was randomly selected within each order from the Global Biodiversity Information Facility. After data cleaning, the final 19962 location will be used for subsequent analyses. The sample size is already large enough to reflect the different distribution temperatures of high- and low-Si plants.<br>To consider the intraspecific variation in leaf phytolith/Si concentrations, a sampling campaign was conducted in China on two typical high-Si plants (wheat and rice) and two low-Si plants (weeping willow and winter jasmine). We collected 475 wheat individuals, 279 rice individuals, 140 weeping willow individuals, and 80 winter jasmine individuals across China. The distribution of the sampling sites is 86.02-126.72 °E and 19.49-45.74 °N. This collection ensures that the sample size is large enough for various analyses and experiments and can well represent the characteristics of different geographical environments in the main production area. At the same time, such collection not only meets research needs but also takes into account considerable human, material, and time costs.<br>In order to identify the most important climatic factors affecting silicon concentration in high silicon plants, we obtained climate variables of sampling points from Resource and Environmental Science Data Center (RESDC, http://www.resdc.cn/DOI) and CHELSA (Table S3), including mean annual temperature (MAT), average ground surface temperature (GST), evaporation (EVP), precipitation (PRE), average pressure (PRS), average relative humidity (RHU), average wind speed (WIN), surface downwelling shortwave flux in air (RSDS), vapor pressure deficit (VPD).<br>Leaf silicon concentrations of 1,826 species and 213 families were obtained from de Tombeur's database, the largest database of leaf silicon concentrations integrating studies from the past 30 years (also containing all the information about silicon from the TRY Plant Trait Database).<br>For the differentiation time of high and low silicon plants, we not only analyzed 17 high- and 28 low-Si families, but considering that Asteraceae, Orchidaceae, Fabaceae, Rubiaceae, and Poaceae are the five largest families of angiosperms, we analyzed each of these families separately to emphasize the applicability of our conclusions.
Data collection	The samples were collected by Zhihao Pang, Lixue Qiu, Li Tan, Wenjuan Li, Enqiang Zhao, Alin Song, Ning Ling, Wei Liu, Hong Pan, Tao Liu, Ling Xiao, Jianping Yang, Jianxiao Wang, Zhuoxi Xiao, Weifeng Xu, Zhenhua Zhang, Gaofei Ge, Ying Zhang, Ping Li, Qi Tao, Xiaozhong Wang, Mujun Ye, Yiyong Zhu, Xiaoling Yang, Peiyuan Cui, Jie Zhang, Zhiyang Jiang, Zhaohui Wang, Jinjia Gan, Jiawei Shi, Liqin Gao, and Zhizhen Ye. The data was recorded in Excel by Zhihao Pang.
Timing and spatial scale	Rice and wheat collections were made from June to October 2021, which matched their harvest seasons. In addition, weeping willow and winter jasmine collections were carried out in June 2024.
Data exclusions	From the 20,000 plant occurrences obtained from GBIF, data with missing latitude and longitude information were eliminated. The final 19,962 occurrences were used for subsequent analysis.
Reproducibility	For the experiments on the effects of high and low temperatures on rice growth, all our replicates were similar to the results reported in the manuscript.
Randomization	Contained in this manuscript are controlled experiments using plants, including comparing the effects of high and low temperatures on plants, and the distribution and evolutionary timing of high and low silicon plants, so no randomization was required.
Blinding	The investigators were blinded to group allocation during data collection and analysis.

Did the study involve field work? ☒ Yes ☐ No

Field work, collection and transport

Field conditions	Rice and wheat collections were carried out from July to October 2021, and weeping willow and winter jasmine collections were carried out in June 2024, with no rainfall in the week prior to sampling.
Location	The distribution of the sampling sites is 86.02-126.72 °E and 19.49-45.74 °N (see Table S3 for details).
Access & import/export	Plant collections have been reported to the local Department of Agriculture. No import or export was involved in this study.
Disturbance	This study had a negligible impact on food production at the sampling sites and caused no disturbance to the agro-ecosystem.

Reporting for specific materials, systems and methods

We require information from authors about some types of materials, experimental systems and methods used in many studies. Here, indicate whether each material, system or method listed is relevant to your study. If you are not sure if a list item applies to your research, read the appropriate section before selecting a response.

Materials & experimental systems

n/a	Involved in the study	
☒	☐	Antibodies
☒	☐	Eukaryotic cell lines
☒	☐	Palaeontology and archaeology
☒	☐	Animals and other organisms
☒	☐	Clinical data
☒	☐	Dual use research of concern
☐	☒	Plants

Methods

n/a	Involved in the study	
☒	☐	ChIP-seq
☒	☐	Flow cytometry
☒	☐	MRI-based neuroimaging

Dual use research of concern

Policy information about dual use research of concern

Hazards

Could the accidental, deliberate or reckless misuse of agents or technologies generated in the work, or the application of information presented in the manuscript, pose a threat to:

No	Yes	
☒	☐	Public health
☒	☐	National security
☒	☐	Crops and/or livestock
☒	☐	Ecosystems
☒	☐	Any other significant area

Experiments of concern

Does the work involve any of these experiments of concern:

No	Yes	
☒	☐	Demonstrate how to render a vaccine ineffective
☒	☐	Confer resistance to therapeutically useful antibiotics or antiviral agents
☒	☐	Enhance the virulence of a pathogen or render a nonpathogen virulent
☒	☐	Increase transmissibility of a pathogen
☒	☐	Alter the host range of a pathogen
☒	☐	Enable evasion of diagnostic/detection modalities
☒	☐	Enable the weaponization of a biological agent or toxin
☒	☐	Any other potentially harmful combination of experiments and agents

Plants

Seed stocks	Rice seeds (Oryza sativa L. cv. Nipponbare) are from the China Rice Data Center of the China National Rice Research Institute (https://www.ricedata.cn/variety/varis/602979.htm).
Novel plant genotypes	This study did not involve novel plant genotypes.
Authentication	This study did not involve authentication.
