# Supplementary material for: Convergent evidence for the temperature-dependent emergence of silicification in terrestrial plants
Source: Nat Commun. 2025 Jan 29;16:1155. doi: 10.1038/s41467-025-56438-0 (PMC11779819; doi:10.1038/s41467-025-56438-0)
Supplement: Supplementary file 4 — Description of Additional Supplementary Files [file 41467_2025_56438_MOESM4_ESM.pdf]

Description of Additional Supplementary Files

Supplementary Data 1

Description: The protein sequences of the top 20 species with the highest homology to Lsi1, 2, 3, and 6 of rice (*Oryza sativa* ssp. japonica).

Supplementary Data 2

Description: The Si concentration in leaves of 1826 species and 213 families.

Supplementary Data 3

Description: Historical climate temperatures (150 Ma ago to present) and geological period names.

Supplementary Code 1

Description: Relationship between the distribution of high- and low-Si clades and temperature.

Supplementary Code 2 Description: Relationship between Si concentration and climate variables in typical high- and low-Si plants in China.

Supplementary Code 3

Description: Analysis of leaf Si concentration in different species and families.
